# Fimbria-Fornix Volume Is Associated With Spatial Memory and Olfactory Identification in Humans

**DOI:** 10.3389/fnsys.2019.00087

**Published:** 2020-01-14

**Authors:** Louisa Dahmani, Blandine Courcot, Jamie Near, Raihaan Patel, Robert S. C. Amaral, M. Mallar Chakravarty, Véronique D. Bohbot

**Affiliations:** ^1^Integrated Program in Neuroscience, McGill University, Montreal, QC, Canada; ^2^Douglas Brain Imaging Center, Department of Psychiatry, McGill University, Montreal, QC, Canada; ^3^Douglas Mental Health University Institute, Department of Psychiatry, McGill University, Montreal, QC, Canada

**Keywords:** spatial memory, navigation, hippocampus, fimbria-fornix, white matter, olfaction

## Abstract

White matter pathways that surround the hippocampus comprise its afferent and efferent connections, and are therefore crucial in mediating the function of the hippocampus. We recently demonstrated a role for the hippocampus in both spatial memory and olfactory identification in humans. In the current study, we focused our attention on the fimbria-fornix white matter bundle and investigated its relationship with spatial memory and olfactory identification. We administered a virtual navigation task and an olfactory identification task to 55 young healthy adults and measured the volume of the fimbria-fornix. We found that the volume of the right fimbria-fornix and its subdivisions is correlated with both navigational learning and olfactory identification in those who use hippocampus-based spatial memory strategies, and not in those who use caudate nucleus-based navigation strategies. These results are consistent with our recent finding that spatial memory and olfaction rely on similar neural networks and structures.

## Introduction

Many people have experienced instances where a smell spontaneously brought back a vivid memory. Therefore, it would not come as a surprise that olfaction could be closely associated with hippocampal-dependent memory, characterized by its vividness and richness in detail (Moscovitch et al., 2005). Olfaction is a vital navigation tool for many species. Many animals use olfactory cues and gradients to locate prey or pups, identify territory, or to go back to previously visited places. Although not crucial for survival, humans are also capable of finding a target location using olfactory cues (Jacobs et al., 2015).

Over the years, numerous studies have investigated spatial memory and its neural correlates. Animal and human studies have found the hippocampus to play a critical role in both spatial memory (O’Keefe and Nadel, 1978; Packard et al., 1989; McDonald and White, 1993; Pigott and Milner, 1993; Maguire et al., 1998; Bohbot et al., 2002, 2004; Hartley et al., 2003; Iaria et al., 2003; Head and Isom, 2010) and olfaction (see Suzuki et al., 2001; Lundström et al., 2011 for reviews; Kjelvik et al., 2012; Wu et al., 2012).

Up until recently, the relationship between spatial memory and olfaction had not been directly investigated. In 2018, we demonstrated the existence of an intrinsic relationship between the two processes (Dahmani et al., 2018). However, this relationship is specific to certain types of navigation but not others. There are two distinct memory systems that can be used for navigation. One of these is the hippocampal memory system, which is critical for the spatial memory strategy. This strategy involves learning precise spatial relationships between landmarks in the environment, so as to form a cognitive map, or mental representation, of one’s environment (O’Keefe and Nadel, 1978). The other memory system involves the caudate nucleus, which is critical for the stimulus-response strategy. This strategy involves learning a set of stimulus-response associations, e.g., a series of motor actions in response to a stimulus (Packard et al., 1989; McDonald and White, 1993). An example of this is route learning, where one must learn to turn left or right in response to various stimuli (e.g., turn left at the gas station). The stimulus-response strategy mainly relies on the caudate nucleus/dorsal striatum (Packard et al., 1989; McDonald and White, 1993; White and McDonald, 2002; Hartley et al., 2003; Iaria et al., 2003; Bohbot et al., 2007; Head and Isom, 2010; Konishi et al., 2013). Importantly, studies from our laboratory showed that gray matter in the hippocampus and caudate nucleus correlates with the respective use of spatial memory and stimulus-response strategies in dual-solution tasks (Iaria et al., 2003; Bohbot and Corkin, 2007; Etchamendy et al., 2012; Konishi and Bohbot, 2013; Konishi et al., 2013; Dahmani and Bohbot, 2015; Dahmani et al., 2018). Using these paradigms to identify individuals’ spontaneous navigation strategies, we showed in a recent report that olfactory identification is associated with faster learning in participants who spontaneously used a spatial memory strategy (spatial learners), but not in those who used a stimulus-response strategy (response learners; Dahmani et al., 2018). This finding was consistent with our hypothesis that olfactory identification would be related to hippocampal-dependent navigation only. In the same study, we measured hippocampal gray matter volume and found it to be positively associated with both olfactory identification and spatial learning, but not with stimulus-response learning.

In the current artice, we turn our attention to white matter. In rodents, many lesion studies have found the fimbria-fornix to be of crucial importance in spatial memory (Olton and Samuelson, 1976; Olton and Papas, 1979; Packard et al., 1989; McDonald and White, 1993, 1995; de Bruin et al., 2001), as it connects the hippocampus to most of its output regions. In contrast, fimbria-fornix lesions do not impair stimulus-response learning (Packard et al., 1989; McDonald and White, 1993; de Bruin et al., 2001). In humans, Iaria et al. (2008) found that hippocampal fractional anisotropy, which is thought to be a measure of structural white matter integrity, was positively associated with spatial learning and memory. However, whether the fimbria-fornix has a dissociable role in human spatial learning and stimulus-response learning is still unknown. Additionally, diffusion magnetic resonance imaging (MRI) measures, including fractional anisotropy, are still under debate, as their association with underlying white matter structure is not clear (Jones et al., 2013; Scholz et al., 2014). In the current study, we investigate the volume of the fimbria-fornix and use a dual-solution virtual navigation task to examine its association with spatial memory and stimulus-response learning. Similar to our previous study (Dahmani et al., 2018), we hypothesized that greater volume of the fimbria-fornix fiber system would be positively associated with navigation and olfactory identification in spatial learners, but not response learners.

## Materials and Methods

### Participants

We tested 60 healthy young adults between the ages of 18 and 35 (mean age = 22.9 ± 3.5; 29 women, 31 men). This represents the same dataset as reported in Dahmani et al. (2018). Participants were excluded if they were not right-handed, if they had a history of neurological or psychiatric disorders, a history of alcohol or drug abuse, or if they suffered a head trauma followed by a loss of consciousness. Three participants did not complete the navigation task (4-on-8 Virtual Maze, described below) and two participants did not undergo MRI scanning. We, therefore, had 55 participants for the navigation and olfaction analyses. The protocol was approved by the local ethics committee of the Douglas Mental Health University Institute. All subjects gave written informed consent in accordance with the Declaration of Helsinki.

### Olfactory Identification

To assess olfactory identification, we administered the Monell Extended Sniffin’ Sticks Identification Test (MONEX-40; Freiherr et al., 2012). This test consists of 40 felt-tip pens, each infused with an odor. The experimenter places each pen under the participant’ nose for one to 2 s and the participant inhales the odor. For each pen, participants are asked to identify the odor from four written choices shown on a screen. The pens were developed to yield at least 35% accuracy across participants (Lundström, personal communication). Thus, in the current study, pens that yielded an overall accuracy that was lower than 35% were excluded from the analysis. Two pens were excluded (warm milk and honey) and the olfactory identification score was based on 38 items. Possible reasons for poor accuracy are degradation of the odor over time, unfamiliarity due to cultural differences, or variability in performance in specific cohorts.

### 4-on-8 Virtual Maze (4/8 VM)

**Figure 1:**
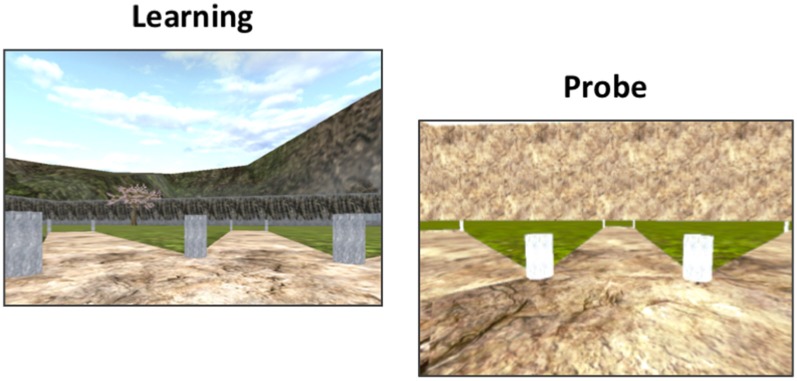
The 4-on-8 Virtual Maze. The 4-on-8 Virtual Maze (4/8 VM) consists in an 8-arm radial maze surrounded by landmarks. In Part 1, four of the paths are blocked and four are open. Participants have to retrieve objects at the end of the open paths. In Part 2, the barriers are removed. Participants have to avoid the paths they visited in Part 1 to retrieve the remaining objects. They can learn the object locations using a spatial memory strategy (e.g., “the path is to the left of the boulder”) or a stimulus-response strategy (“From the starting position, I have to take the path straight ahead and then skip a path on the right”). Once participants learn the task to criterion, they are taken to a probe stage, where a wall is raised around the maze that hides the landmarks. People who used a spatial memory strategy during learning make more errors than people who used stimulus-response strategies, as they can no longer use landmarks to find the target paths. At the end of the task, participants have to draw a map of the maze and are administered a verbal report, which serves to determine the strategy they used as well as the number of landmarks they used (e.g., “I used the rock and the tree to find the objects”) and noticed (e.g., “I saw a mountain but I did not use it”).

The 4/8 VM (Figure 1) was developed using Unreal Tournament 2003 (Epic Games, Raleigh, NC, USA). The task was adapted from a maze task originally used in rodents (Olton and Samuelson, 1976; Packard et al., 1989) and consists in a radial maze surrounded by a rich landscape with proximal and distal landmarks. The maze is made of a central platform with eight paths branching out around it. There are two parts to each learning trial:

- Part 1: Out of the eight paths, four are blocked and four are accessible. Participants are instructed to visit the accessible paths, to retrieve objects at the end of the paths (which are not visible from the central platform), and to memorize their location. When ready, participants are taken to Part 2.
- Part 2: Here, we remove the barriers to make all the paths accessible. Participants are asked to avoid the paths they just visited in order to retrieve the remaining objects. Participants can use either a spatial memory strategy, by learning the spatial relationships between landmarks in the environment and the target paths (e.g., “there is an object to the left of the tree and one to the right of the boulder”), or they can use a stimulus-response strategy, by learning a series of motor actions in response to a stimulus (e.g., “from the starting position, I have to go straight ahead and then skip the path on the right”).

A minimum of three and a maximum of eight learning trials are administered. The location of the objects does not change throughout the task except in the second trial. Participants are required to find the objects in Part 2 without making errors in at least one of the trials in order to reach the learning criterion. Once this is achieved, we administer a probe trial. Part 1 of the probe trial is the same as above. In Part 2, a wall is erected around the radial maze, blocking the landmarks from the participants’ view. Participants who used a spatial memory strategy (“spatial learners”) to memorize the object locations make more errors than participants who used a stimulus-response strategy (“response learners”), as they can no longer rely on the landmarks to find the paths containing the objects (Iaria et al., 2003). The probe trial is followed by a normal trial. Once the task is done, we conduct a verbal report, where we ask participants to describe how they solved the task. We used the verbal report to categorize participants into spatial or response learners. If participants mentioned learning the location of the pathways relative to several landmarks, they were categorized as spatial learners. If they mentioned using a counting strategy or a sequence starting from the start position of a single position demarked by one landmark, they were categorized as response learners. We previously showed that spontaneous strategies, i.e., strategies used in the very first trial, are associated with increased functional magnetic resonance imaging (fMRI) blood oxygenation-level-dependent (BOLD) activity and gray matter in our regions of interest (Iaria et al., 2003; Bohbot et al., 2007). The verbal report is also used to determine how many landmarks participants noticed in the environment and how many landmarks they used on average throughout the learning trials.

The dependent variables are number of trials to criterion, the average number of errors on Part 2 of the learning trials (these provide two slightly different measures of navigational learning), spontaneous navigation strategy, number of landmarks noticed the average number of landmarks used during the learning phase of the task, and probe errors.

### Neuropsychological Assessment

To assess potential differences between spatial and response learners in neuropsychological status, we administered the following neuropsychological tests: the Rey Auditory Verbal Learning Task (Rey, 1941) to evaluate verbal memory, the Rey-Osterrieth Complex Figure (Meyers and Meyers, 1995) to assess visuospatial memory, and the Test of Non-verbal Intelligence-3 (Brown et al., 1997) to assess non-verbal intelligence. Spatial and response learners did not differ on these tests (all Bootstrap BCa 95% CI crossed 0).

### MRI Data Acquisition

We acquired anatomical MRI data at the Douglas Cerebral Imaging Centre, using a 3 Tesla Siemens Magnetom Trio scanner equipped with 12-channel array coil. We immobilized participants’ heads using support cushions. A localizer scan was first acquired, followed by a T1-weighted 3D MPRAGE anatomical scan with 192 contiguous 1 mm slices in the sagittal plane (TR = 2,300 ms; TE = 2.98 ms; flip angle = 9; the field of view = 256 mm^2^), resulting in an acquisition time of 9:14 min.

### MRI Data Analysis

**Figure 2:**
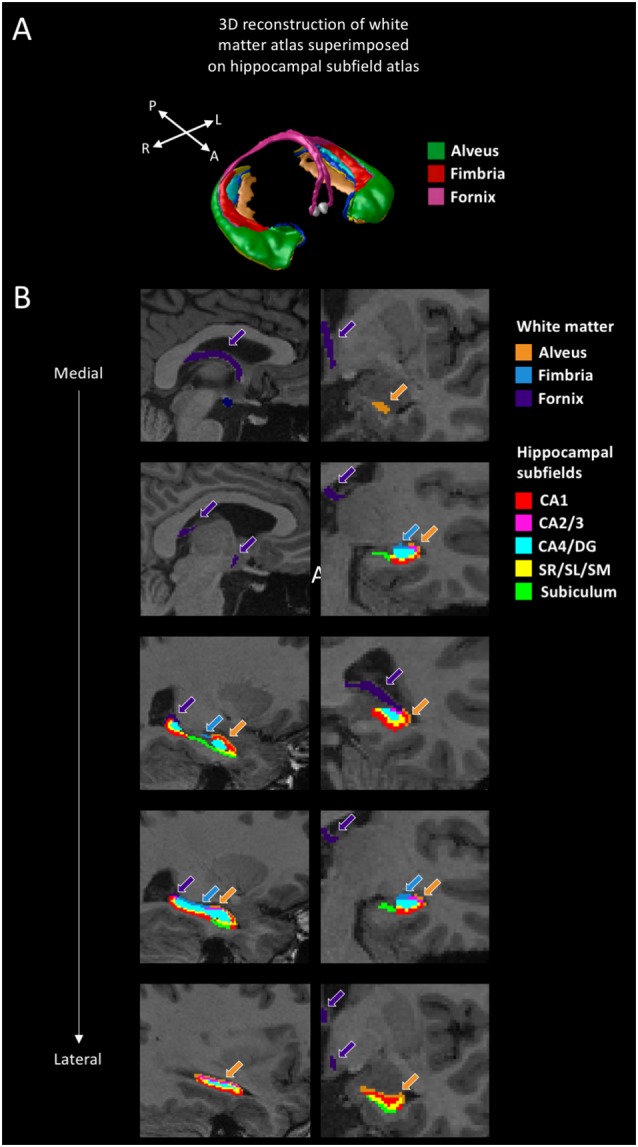
White matter segmentation. **(A)** 3D reconstruction of the white matter atlas (Amaral et al., 2018), showing the alveus, fimbria, and fornix, superimposed on the hippocampal subfield atlas (Pipitone et al., 2014). **(B)** Segmentations of the alveus (in orange), fimbria (in blue), and fornix (in purple) are shown for a representative participant. The other colors represent the various hippocampal subfields (not discussed in this article but see Dahmani et al., 2018). Sagittal views are shown on the left, coronal views are shown on the right. A: anterior; P: posterior; R: right; L: left; CA: cornu ammonis; DG: dentate gyrus; SR/SL/SM: stratum radiatum, lacunosum, and moleculare.

The Multiple Automatically Generated Templates (MAGeT) Brain tool was used to automatically segment extra-hippocampal white matter fiber bundles (Amaral et al., 2018; Figure 2A). This tool was developed to use a small set of high-quality atlases that were manually segmented as input. Participants’ structural scans were processed with N4 intensity correction (Tustison et al., 2010). We then applied a head mask to ameliorate registration before running MAGeT-Brain. The Winterburn Atlas (Winterburn et al., 2013) was adapted to include the extra-hippocampal white matter bundles and is comprised of five manually-segmented brains (Amaral et al., 2018). In MAGeT-Brain, a library of 21 templates (Pipitone et al., 2014) is used to bootstrap each individual’s segmentation. The templates were chosen by first segmenting all of the samples using the five manually segmented brains and then selecting the ones presenting the best registrations in order to increase registration quality for the full sample analysis. We used non-linear atlas-to-template registration to segment and label each template, which resulted in a unique delineation of the subfields for each individual template. The bootstrapping yields 105 candidate labels for each individual (5 atlases × 21 templates), which are subsequently fused through a voxel-wise majority vote to output one final segmentation (Figure 2B). We used the Automatic Normalization Tools (ANTS) registration technique for non-linear registration^1^. The extra-hippocampal white matter bundles that we inspected included the fimbria, fornix, and alveus of the hippocampus. Together, these form the fimbria-fornix white matter bundle. We visually inspected each output segmentation for quality control, which all segmentations passed.

### Analysis

We used SPSS Statistics 20 (IBM) for data analysis. We performed partial correlations between our olfaction and 4/8 VM variables and white matter volumes. Sex was used as a covariate because men have on average a larger brain than women. We used bootstrapped bias-corrected and accelerated 95% confidence intervals (Bootstrap BCa 95% CI; Field, 2009) to determine significance. Bootstrapping is a resampling method that uses a sample dataset and simulates 1,000 datasets from this set by resampling with replacement. This method is useful in that it inherently corrects for multiple comparisons (Westfall and Young, 1993; Field et al., 2012). Confidence intervals are an estimation of the population’s true value, which makes them both more accurate and more robust than *p*-values (Rothman, 1978; Poole, 2001; Greenland et al., 2016), especially when bootstrapping methods are used (Westfall and Young, 1993). Resampling methods also offer the advantage of estimating Type I and Type II error rates more precisely than standard *p*-value adjustment methods (Field et al., 2012). It is, therefore, unnecessary to further correct for multiple comparisons. Another advantage of bootstrapping methods is that they are non-parametric, and thus do not require to transform the data when it is not normally distributed (Haukoos and Lewis, 2005; Field et al., 2012). We used one-tailed confidence intervals when analyses were hypothesis-driven. We first investigated the overall right and left fimbria-fornix white matter volumes. These were determined by calculating the sum of the fimbria, fornix, and alveus of the hippocampus, for each hemisphere. If no significant correlation was found between overall fimbria-fornix volumes and our measures of interest, then we investigated the sub-regions (fimbria, fornix, and alveus of the hippocampus) separately to see if any effect existed at a smaller scale.

## Results

Using participants’ 4/8 VM verbal report, we categorized 23 participants as spatial learners and 32 participants as response learners. We performed two sets of analyses: we looked at the associations between: (1) navigation and white matter volumes; and (2) olfaction and white matter volumes. We hypothesized that, as with our previous behavioral and structural neuroimaging results (Dahmani et al., 2018), only spatial learners would show an association between fimbria-fornix volume, olfaction, and navigation. Table 1 shows the correlation coefficients and bootstrap BCa 95% CI of the correlations between white matter volumes and navigation and olfaction variables for spatial and response learners, with sex as a covariate.

**Table 1:** Partial correlations between variables of interest and white matter bundle volumes for spatial and response learners.

	Spatial learners	Response learners	*r* (CI)	*r* (CI)
**Average navigational learning errors**		
R fimbria-fornix	−0.40 (−0.66, −0.15)*	0.10 (−0.26, 0.37)
R fimbria	−	−0.09 (−0.42, 0.24)
R fornix	−	0.05 (−0.34, 0.41)
R alveus	−	0.23 (−0.16, 0.48)
L fimbria-fornix	−0.19 (−0.52, 0.10)	0.04 (−0.27, 0.29)
L fimbria	−0.31 (−0.60, 0.004)^†^	0.08 (−0.23, 0.32)
L fornix	−0.21 (−0.52, 0.08)	0.02 (−0.25, 0.25)
L alveus	0.003 (−0.40, 0.33)	0.04 (−0.32, 0.33)
**Number of trials to criterion**		
R fimbria-fornix	−0.09 (−0.42, 0.25)	0.08 (−0.19, 0.31)
R fimbria	−0.35 (−0.57, −0.15)*	−0.13 (−0.40, 0.14)
R fornix	−0.18 (−0.48, 0.14)	0.06 (−0.27, 0.31)
R alveus	0.17 (−0.15, 0.45)	0.20 (−0.16, 0.63)
L fimbria-fornix	0.05 (−0.26, 0.35)	0.01 (−0.28, 0.34)
L fimbria	−0.17 (−0.49, 0.13)	0.06 (−0.20, 0.39)
L fornix	0.06 (−0.25, 0.36)	−0.05 (−0.40, 0.31)
L alveus	0.09 (−0.16, 0.34)	0.09 (−0.19, 0.32)
**Olfactory identification**		
R fimbria-fornix	0.24 (−0.10, 0.55)	−0.12 (−0.37, 0.20)
R fimbria	0.41 (0.08, 0.68)*	0.18 (−0.13, 0.47)
R fornix	0.07 (−0.28, 0.41)	−0.24 (−0.50, 0.14)
R alveus	0.26 (−0.05, 0.69)	0.02 (−0.28, 0.31)
L fimbria-fornix	0.14 (−0.16, 0.43)	−0.03 (−0.35, 0.25)
L fimbria	0.01 (−0.37, 0.39)	0.03 (−0.28, 0.34)
L fornix	0.20 (−0.18, 0.52)	0.06 (−0.31, 0.35)
L alveus	0.01 (−0.33, 0.42)	−0.19 (−0.41, 0.05)

Correlation coefficients (*r*) are shown with bootstrap bias-corrected and accelerated 95% confidence intervals in brackets. All correlations were covaried with sex. R: Right; L: Left. *Indicates correlations that are significant (bootstrap bias-corrected and accelerated 95% confidence intervals do not cross 0). ^†^Denotes marginally significant correlations (confidence intervals barely cross 0).

### Navigation

**Figure 3:**
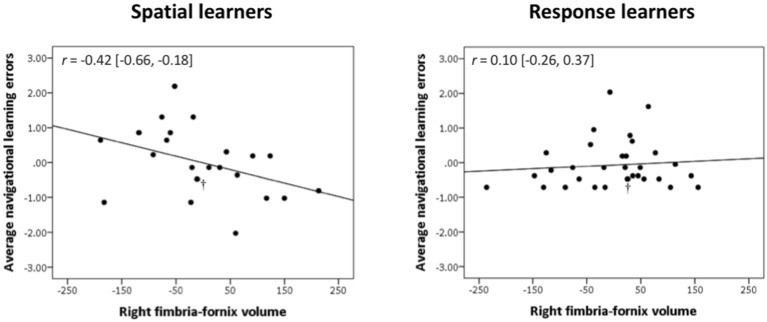
Right fimbria-fornix volume is associated with fewer errors during navigational learning in spatial learners, but not response learners. Within spatial learners, average navigational learning errors correlate negatively with right fimbria-fornix volume (shown on the left), *r* = −0.42 [Bootstrap BCa 95% CI (−0.66, −0.18)], but not with left fimbria-fornix volume (Bootstrap BCa 95% CI cross 0). Within the left fimbria-fornix sub-regions, average navigational learning errors show a marginally significant negative correlation with the left fimbria, *r* = −0.31 [Bootstrap BCa 95% CI (−0.60, 0.004)], but not with the other sub-regions (Bootstrap BCa 95% CI cross 0). Within response learners, average navigational learning errors do not correlate with left or right fimbria-fornix volume (shown on the right is the right fimbria-fornix volume correlation for comparison, *r* = 0.10 [Bootstrap BCa 95% CI (−0.26, 0.37)], or any of their sub-regions (Bootstrap BCa 95% CI cross 0). These results indicate that more efficient spatial learning is mainly associated with a greater volume of the right fimbria-fornix fiber system. Stimulus-response learning does not show any correlations with the left or right fimbria-fornix system. †Indicates two overlapping data points.

In spatial learners, we found that faster learning correlates with fimbria-fornix white matter volume: there was a significant negative correlation between right fimbria-fornix volume and average navigational learning errors [*r* = −0.40, Bootstrap BCa 95% CI (−0.66, −0.15; Figure 3, left)], and between right fimbria volume and number of trials to criterion [*r* = −0.35, Bootstrap BCa 95% CI (−0.57, −0.15)]. Additionally, there was a marginally significant negative correlation between left fimbria volume and average navigational learning errors [*r* = −0.31, Bootstrap BCa 95% CI (−0.60, 0.004)]. Average navigational learning errors did not significantly correlate with the left fimbria-fornix volume, although the correlation was in the hypothesized direction [*r* = −0.19, Bootstrap BCa 95% CI (−0.52, 0.10)]. Thus, faster learning in spatial learners is predominantly associated with the right fimbria-fornix.

Response learners showed no significant associations between fimbria-fornix volumes and navigational learning [all Bootstrap BCa 95% CIs crossed 0; e.g., average navigational learning errors and right fimbria-fornix volume, *r* = 0.10, Bootstrap BCa 95% CI (−0.26, 0.37; Figure 3, right)]. There were also no significant associations with sub-regions of the left or right fimbria-fornix (all Bootstrap BCa 95% CIs crossed 0).

Spatial and response learners did not show any correlations between fimbria-fornix volumes and either number of landmarks noticed/used or probe errors (all Bootstrap BCa 95% CIs crossed 0).

### Olfactory Identification

**Figure 4:**
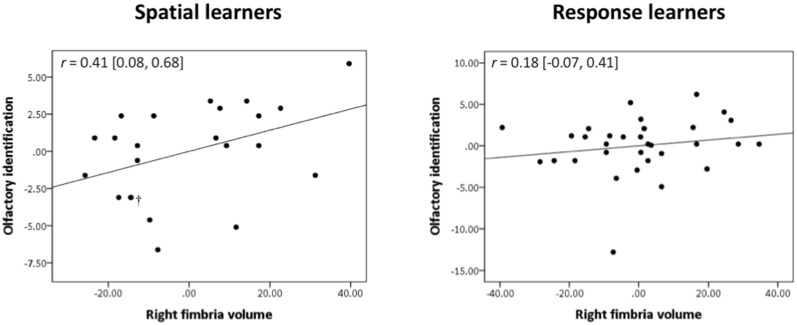
Olfactory identification correlates positively with right fimbria volume within spatial learners only. Within spatial learners, olfactory identification correlates positively with right fimbria volume (shown on the left), *r* = 0.41, Bootstrap BCa 95% CI (0.08, 0.68), but not with the left fimbria-fornix or any of its sub-regions (Bootstrap BCa 95% CI cross 0). Within response learners, olfactory identification does not correlate positively with either left or right fimbria-fornix volume or any of their sub-regions {shown on the right is the association with right fimbria volume for comparison, *r* = 0.18 [Bootstrap BCa 95% CI (−0.07, 0.41)]; all other Bootstrap BCa 95% CI cross 0}. These results indicate that olfactory identification correlates positively with a sub-region of the right fimbria-fornix fiber system in those who use hippocampal-dependent navigation strategies only. †Indicates two overlapping datapoints.

Spatial learners showed a significant positive correlation between olfactory identification and volume of the right fimbria [*r* = 0.41, Bootstrap BCa 95% CI (0.08, 0.68; Figure 4, left)]. They did not show a significant correlation between olfactory identification and right [*r* = 0.24, Bootstrap BCa 95% CI (−0.10, 0.55)] or left [*r* = 0.14, Bootstrap BCa 95% CI (−0.16, 0.43)] fimbria-fornix volumes, or with any sub-regions of the left fimbria-fornix (all Bootstrap BCa 95% CIs crossed 0).

In response learners, there were no associations between olfactory identification and right [*r* = −0.12, Bootstrap BCa 95% CI (−0.37, 0.20)] or left [*r* = −0.03, Bootstrap BCa 95% CI (−0.35, 0.25)] fimbria-fornix volumes. There were also no correlations with any of the fimbria-fornix sub-region volumes (all Bootstrap BCa 95% CIs crossed 0; e.g., right fimbria: Figure 4, right).

## Discussion

In the current study, we examined whether the fimbria-fornix has a dissociable role in spatial learning and stimulus-response learning in humans. We found fimbria-fornix volume to be positively associated with spatial memory. As hypothesized, there is no association between fimbria-fornix volume and stimulus-response learning, further strengthening the idea that these two forms of navigation rely on separate neural networks (O’Keefe and Nadel, 1978; Packard et al., 1989; McDonald and White, 1993). Similarly, within spatial learners, olfactory identification shows positive correlations with the fimbria-fornix, which is not the case within response learners.

These findings are consistent with our previous study (Dahmani et al., 2018), where we found both spatial learning and olfactory identification to be associated with hippocampal gray matter volume, while there were no associations between stimulus-response learning and hippocampal volume.

In terms of lateralization, in our previous report we found an association between spatial memory, olfactory identification, and right hippocampal volume (Dahmani et al., 2018). Our white matter findings are concordant, as the effects we found predominantly involved the right fimbria-fornix fiber system. This is also consistent with a right-sided hippocampal lateralization for spatial memory and olfactory identification often reported in the literature (Habib and Sirigu, 1987; Zatorre and Jones-Gotman, 1990; Zatorre et al., 1992; Jones-Gotman and Zatorre, 1993; Barrash, 1998; Bohbot et al., 1998; Savic et al., 2000; Burgess, 2002; Frasnelli et al., 2010; Kjelvik et al., 2012; Smitka et al., 2012).

The positive association between olfactory identification and the right fimbria-fornix bundle was observed in spatial learners, but not response learners, a pattern of results that reflects that of our previous study (Dahmani et al., 2018). These findings indicate that the fimbria-fornix is involved in olfactory identification in individuals who spontaneously use the hippocampal memory system. The hippocampus itself is reported to be involved in olfactory identification in approximately half of the studies in the literature, while the other half does not report such involvement (Suzuki et al., 2001; Kjelvik et al., 2012, 2014; Seubert et al., 2013; Smitka et al., 2012; Segura et al., 2013). This inconsistency may be a product of inter-individual variability in hippocampal involvement. The fact that we found a correlation between olfactory identification and fimbria-fornix white matter among spatial learners but not response learners indicates that it may be useful to identify individuals’ spontaneous navigation strategies in order to capture this inter-individual variability. We speculated in Dahmani et al. (2018) that response learners may more heavily rely on other nodes of the olfactory network, or that they may use a different neural network for olfactory processing. There is a lot of evidence that olfaction is influenced by top-down processing (Zatorre et al., 2000; Finkel et al., 2001; Gottfried and Dolan, 2003; de Araujo et al., 2005; Grabenhorst and Rolls, 2010; Ferdenzi et al., 2017) and cognitive strategies may influence the neural networks underlying it (Karunanayaka et al., 2014). Future studies should seek to elucidate the neural networks involved in olfactory identification using functional connectivity methods and to determine whether spatial and response learners differ in the way they process olfactory information.

Our findings may have implications for aging research. Both olfaction and spatial memory are impaired early on in cognitive aging and Alzheimer’s disease (Henderson et al., 1989; Pai and Jacobs, 2004; Tu and Pai, 2006; Wilson et al., 2007, 2009; Devanand et al., 2008, 2015; Head and Isom, 2010; Stanciu et al., 2014; Allison et al., 2016; Lafaille-Magnan et al., 2017). Atrophy of the olfactory bulb, olfactory tract, entorhinal cortex, and hippocampus is also observed early in the disease (Pearson et al., 1985; Ferreyra-Moyano and Barragan, 1989; Talamo et al., 1989; Fox et al., 1996; Kaye et al., 1997; den Heijer et al., 2006). Our results suggest that dissociating individuals based on navigation strategies (spatial vs. response) combined with measuring errors during navigational learning and olfactory identification may be more sensitive to variations in hippocampal volume and hippocampal network white matter integrity, compared to looking at navigational learning errors or olfactory identification alone. It would be of interest to pursue this question in a future line of research.

In summary, our finding that fimbria-fornix volume is associated with spatial learning and olfactory identification is in line with our previous results (Dahmani et al., 2018). Not only are these two processes behaviorally linked, but they also share many neuroanatomical substrates (Dahmani et al., 2018). In a comprehensive review, Jacobs et al. (2015) describes that the size of the olfactory bulb, a primary olfactory area, covaries with hippocampal size in many mammals (Reep et al., 2007) and with navigational demand, according to factors such as home range size (Gittleman, 1991) and predatory strategy (Reep et al., 2007). Taken together, these pieces of evidence are consistent with our findings that spatial memory and olfaction are closely linked.

## Data Availability Statement

The raw data supporting the conclusions of this article will be made available by the authors, without undue reservation, to any qualified researcher.

## Ethics Statement

The studies involving human participants were reviewed and approved by the Institutional Review Board of the Douglas Mental Health University Institute. The patients/participants provided their written informed consent to participate in this study.

## Author Contributions

LD and VB designed the study. JN, LD, and VB devised the MRI scanning protocol. LD performed the study, analyzed the data, and wrote the manuscript. BC, JN, and RP assisted with neuroimaging data preprocessing. RP, RA, and MC assisted with the MAGeT-Brain pipeline. VB supervised the study. All authors edited the manuscript.

## Conflict of Interest

The authors declare that the research was conducted in the absence of any commercial or financial relationships that could be construed as a potential conflict of interest.

## Footnotes

^1^https://github.com/vfonov/mincANTS
